# The Role of the Ecotoxicology Applied to Seafood as a Tool for Human Health Risk Assessments Concerning Polycyclic Aromatic Hydrocarbons

**DOI:** 10.3390/ijerph19031211

**Published:** 2022-01-22

**Authors:** Julia Vianna de Pinho, Paloma de Almeida Rodrigues, Ivelise Dimbarre Lao Guimarães, Francielli Casanova Monteiro, Rafaela Gomes Ferrari, Rachel Ann Hauser-Davis, Carlos Adam Conte-Junior

**Affiliations:** 1 Center for Food Analysis (NAL), Technological Development Support Laboratory (LADETEC), Federal University of Rio de Janeiro (UFRJ), Cidade Universitária, Rio de Janeiro 21941-598, RJ, Brazil; juliadepinho@hotmail.com (J.V.d.P.); ivelisedlguimaraes@gmail.com (I.D.L.G.); fran_casanovam@hotmail.com (F.C.M.); rafaelaferrari@yahoo.com.br (R.G.F.); carlosconte@hotmail.com (C.A.C.-J.); 2 National Institute of Health Quality Control, Oswaldo Cruz Foundation, Rio de Janeiro 21040-900, RJ, Brazil; 3 Graduate Program in Sanitary Surveillance (PPGVS), National Institute of Health Quality Control (INCQS), Oswaldo Cruz Foundation (FIOCRUZ), Rio de Janeiro 21040-900, RJ, Brazil; 4 Laboratory of Advanced Analysis in Biochemistry and Molecular Biology (LAABBM), Department of Biochemistry, Federal University of Rio de Janeiro (UFRJ), Cidade Universitária, Rio de Janeiro 21941-909, RJ, Brazil; 5 Graduate Program in Veterinary Hygiene (PPGHV), Faculty of Veterinary Medicine, Fluminense Federal University (UFF), Vital Brazil Filho, Niteroi 24220-000, RJ, Brazil; 6 Agrarian Sciences Center, Department of Zootechnics, Federal University of Paraiba, Areias 51171-900, PB, Brazil; 7 Environmental Health Assessment and Promotion Laboratory, Instituto Oswaldo Cruz, Fundação Oswaldo Cruz, Rio de Janeiro 21040-360, RJ, Brazil; rachel.hauser.davis@gmail.com; 8 Graduate Program in Food Science (PPGCAL), Institute of Chemistry (IQ), Federal University of Rio de Janeiro (UFRJ), Cidade Universitária, Rio de Janeiro 21941-909, RJ, Brazil; 9 Graduate Program in Chemistry (PGQu), Institute of Chemistry (IQ), Federal University of Rio de Janeiro (UFRJ), Cidade Universitária, Rio de Janeiro 21941-909, RJ, Brazil

**Keywords:** PAH, petroleum derivates, organic compounds, marine ecosystem, toxicity assessments, environmental health, marine biota, fish products, mussels

## Abstract

Background: Polycyclic aromatic hydrocarbons (PAHs) are persistent pollutants routinely detected in aquatic ecosystems. It is, therefore, necessary to assess the link between deleterious marine biota PAH effects, especially in commercialized and consumed animals, environmental health status, and potential human health risks originating from the consumption of contaminated seafood products. Thus, this review seeks to verify the relationships of ecotoxicological studies in determining effect and safety concentrations on animals routinely consumed by humans. Methods: A total of 52 published studies between 2011 and 2021, indexed in three databases, were selected following the PICO methodology, and information on test animals, evaluated PAH, and endpoints were extracted. Results: Benzo(a)pyrene and phenanthrene were the most investigated PAHs in terms of biomarkers and test organisms, and mussels were the most evaluated bioindicator species, with an emphasis on reproductive responses. Furthermore, despite the apparent correlation between environmental PAH dynamics and effects on aquatic biota and human health, few assessments have been performed in a multidisciplinary manner to evaluate these three variables together. Conclusions: The links between human and environmental sciences must be strengthened to enable complete and realistic toxicity assessments as despite the application of seafood assessments, especially to mussels, in bioassays, the connection between toxicological animal responses and risks associated with their consumption is still understudied.

## 1. Introduction

Human, environmental, and animal health are all intrinsically correlated according to the One Health concept [1]. This concept, coined in 2004 through the integration of human medicine and veterinary medicine, seeks to incorporate environmental health bases to establish links between ecosystem effects and the triggering of conditions that affect human, animal, and environmental health [2]. Within this view, it becomes clear that multidisciplinary assessments are a valuable tool in the environmental and Public Health fields. As such, human health risk assessments concerning contaminated seafood consumption should not be dissociated from animal health risk assessments. In this context, increasing chemical pollution levels worldwide have significantly increased concerns regarding seafood contamination.

Polycyclic aromatic hydrocarbon (PAH) contamination in marine aquatic environments, in particular, is of significant concern as these compounds are highly persistent and result in deleterious contamination effects that pose potential risks to both animal and human health [3,4,5,6].

**Figure 1:**
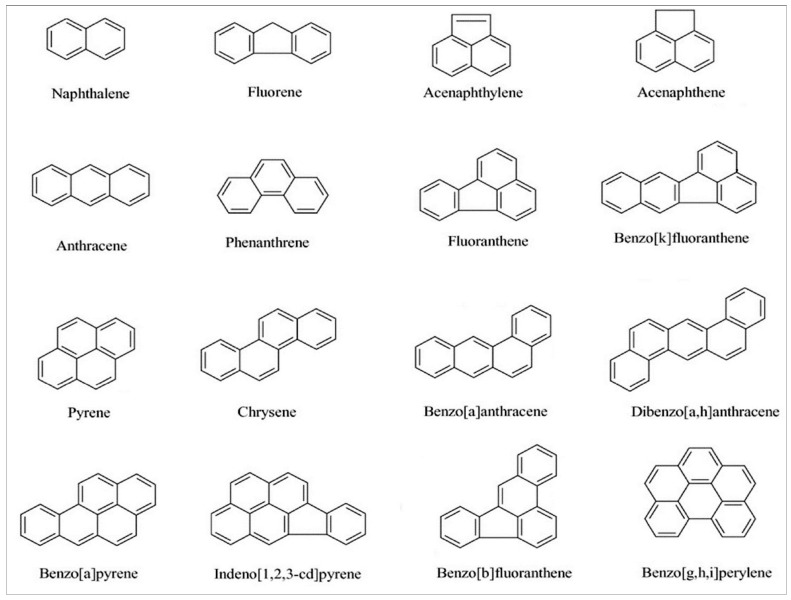
Chemical structure of the 16 priority polycyclic aromatic hydrocarbons according to the United States Environmental Protection Agency.

Different human activities such as fuel, wood and coal burning, industrial effluents discharges, oil extraction, and ship traffic [7,8,9,10,11] comprise the primary PAH sources, responsible for the input of about 200 compounds belonging to the PAH group into the environment [12]. High PAH concentrations have been detected in sediments, rivers, and fishes [3]. Moreover, these compounds are known to biomagnify along the food chain [13,14], reaching humans through seafood consumption, potentially resulting in several deleterious health effects to both animals and humans, such as decreased immune function, kidney and liver damage, as well as carcinogenicity and genotoxicity [15]. Despite their global distribution [16], 16 of these substances, in particular, require priority control due to their high toxicity (Figure 1).

**Figure 2:**
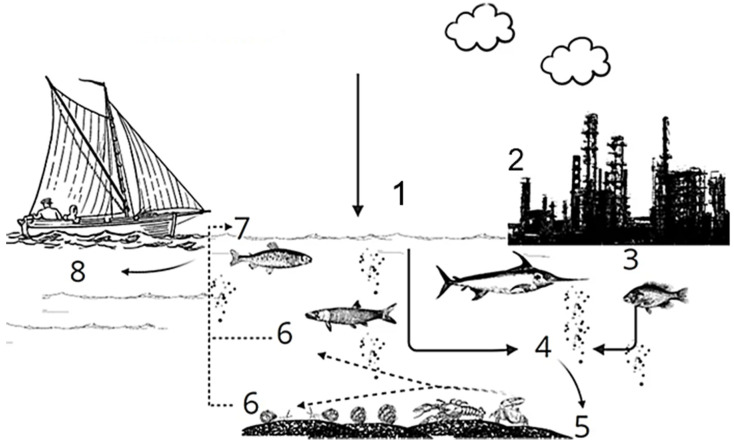
PAH dynamics in marine ecosystems. (1) Atmospheric PAH deposition and (2) oil spills (3) that may contaminate aquatic environments, (4) followed by adsorption to suspended particulate matter, and (5) sedimentation processes. (6) Benthonic organisms are exposed to PAH through the dietary route, (7) which then bioaccumulate and biomagnify throughout the food chain, (8) reaching high levels in larger fish consumed by humans, characterizing a potential human health risk.

Due to their high molecular stability, PAHs tend to remain and circulate between environmental compartments for extended periods of time [17,18,19]. However, they present low or no solubility in water, which, alongside their resistance to biodegradation, makes them susceptible to adsorption onto suspended particles and/or sedimentation [20], creating a direct exposure route to benthic biota (Figure 2). 

As PAHs display the ability to interact with lipid cell membranes, due to their lipophilicity, they display bioaccumulative properties, and high levels have been reported for different trophic niches, i.e., zooplankton, mussels, fish, aquatic, and terrestrial mammals [21,22,23]. Several studies indicate negative toxic effects following PAH exposure in several taxonomic groups, which may, in turn, alter ecosystem dynamics as different organismic responses to intoxication, whether physiological, morphological, or biochemical, may indicate links between contaminants and ecological effects [24]. In this regard, in addition to determining the toxic effects of chemical substances on aquatic organisms themselves, human health risks should also be considered [25], as contaminated seafood items are considered one of the most severe human health hazards. 

As aforementioned, the One Health concept indicates that human and animal health and environmental conditions are significantly interrelated [2]. However, obtaining an equilibrium among these three parameters is still a challenge, which can be adequately assessed by employing interdisciplinary fields in evaluations, such as ecotoxicology and risk assessments [2,26]. While ecotoxicology makes it possible to assess the impacts of contaminants on organisms in advance by conducting controlled tests with standardized test organisms, risk assessments aim to determine whether the level of exposure to a certain contaminant to which a population is exposed to for their entire lives, considering the average life expectancy of the exposed population, is considered safe, by assessing xenobiotic concentrations, bioavailability, intoxication pathways, and degree of toxicity, and are paramount in developing strategies to improve public health status [26]. Ecotoxicology can, for example, address a series of potential final effects, termed endpoints, such as the lethal concentration 50% (LC50) of a certain substance, capable of killing 50% of exposed individuals, or a 50% effect concentration (EC50), which assesses changes in parameters other than death in exposed organisms, such as growth and reproduction. The application of different assays, however, evaluating more primary endpoints such as biochemical, cytological, and histological effects, has been highlighted in recent years, allowing for assessments on interaction effects between contamination sources [23]. Concerning human risk assessments, the hazard quotient (HQ) considers factors such as the frequency of contaminated food consumption, exposure duration, contaminant concentrations, and maximum permissible reference doses [27,28]. Furthermore, due to the carcinogenic effects attributed to PAHs, the USA EPA (1991) also establishes a cancer risk assessment model based on the average lifespan of exposed humans and estimates the probability of an individual developing cancer over their life history [29].

In this context, this study aims to verify the applicability of ecotoxicological data concerning contaminated seafood in human health risk assessments by employing a systematic review.

## 2. Materials and Methods

### 2.1. Focus Question

The population, intervention, and comparisons were defined according to the PICO method. The central question of this study was established as “Do published ecotoxicological studies on PAHs, employing marine biota, enable the assessment of human health risks associated with contaminated seafood consumption?” This was answered through sub-items such as “Which organisms have been used in toxicity tests and under which exposure levels?”, “Which parameters and endpoints were evaluated in these studies?”, “What is the degree of risk concerning human health by the consumption of marine biota, and is this risk directly related to the effects observed in contaminated animals?”

### 2.2. Information Sources

The applied descriptors employed in the data search were selected from the DECS/MeSH platform. The search was carried out between August and September 2021 at the “Web of Science”, “PubMed”, and “Embase” databases. To direct the inquiry, four “Search Components” were defined:

- Search Component 1 (SC1): Population: “Arthropoda” OR “Arthropods” OR “Crustacean” OR “fish” OR “Fish products” OR “Fish culture” OR “Clams” OR “Mussel” OR “Bivalvia.”
- Search Component 2 (SC2): “Ecotoxicology” OR “Environmental toxicology” OR “Effects” OR “Toxicity” OR “Toxicology” OR “Bioassay” OR “Lethal concentration” OR “Effect concentration” OR “Environmental health” OR “Marine toxicity” OR “Aquatic toxicity” OR “Human health” OR “Human risk”.
- Search Component 3 (SC3): “pah” OR PAH OR “Polycyclic aromatic hydrocarbons” OR “Polynuclear aromatic hydrocarbon.”
- Search Component 4 (SC4): AND NOT (“detection” OR “collected”)

Following four sequential stages, two authors (J.V.A.d.P. and P.d.A.R.) first conducted a preliminary selection of the identified abstracts and paper titles independently. Abstracts were removed if the papers did not investigate associations between the search components. Articles whose abstracts suggested applying bioassays using water, oil, or sediment contaminated by PAHs or that performed the quantification of these elements in animals sampled from potentially impacted environments were also removed. Studies whose abstracts did not mention animals or that demonstrate the application of mathematical models were also removed.

The search was limited to English, and publishing dates were set between 2011 and 2021. Editorials, letters, reviews, mini-reviews, and M.Sc. dissertations and Ph.D. theses were excluded. 

Considering that the use of the descriptors “PAH” and “Risk” could be linked to the evaluation of oil exploration with metal contamination and the general definition of dangers from PAH poisoning without actually analyzing these compounds or performing risk assessments following application models (i.e., USEPA models), some studies were excluded due to the following:

- Ecological risk assessments based on PAH distributions in the aquatic or marine environment;
- Oil spills or oil products and their effects on biota;
- Epidemiological studies to determine the incidence/prevalence of diseases associated with the consumption of contaminated fish;
- Development or applications risk assessment methodologies disregarding the effects presented by investigated organisms (animals, plants, and humans) subjected to specific PAH exposure concentrations.

In addition, two screenings were conducted for article selection and extraction to ensure the removal of studies that did not meet the established criteria, such as the specific application of a PAH compound or the use of fish products. In the first selection stage, article suitability was evaluated through reading the abstracts and those fitting the following inclusion criteria:

- Assessments concerning PAH effects on test organisms under controlled conditions;
- Assessments indicating the tested organism, the PAH, test/effect concentration, and the evaluated endpoint;
- Evaluations concerning animals sampled from the environment or kept in the laboratory for laboratory exposures;
- Indications of assay time and affected biomarkers or physiological or morphological alterations.

In the second screening (extraction), the articles selected in the first stage were re-evaluated in their entirety to confirm the initial selection and restrict the analysis to seafood interest following the previously mentioned exclusion criteria.

The results are reported in agreement with the Preferred Reporting Items for Systematic Review and Meta-Analyses statement by the SR management StArt tool. After selection, the end analyses of the results were plotted as column graphs using the ggplot2 package, available in the R software (version 4.0.4) (R Foundation for Statistical Computing, Vienna, Austria) [30].

Possible bias sources include inclusion/exclusion criteria, the chosen database, date, language, number of articles, and article types selected for this study.

## 3. Results

**Figure 3:**
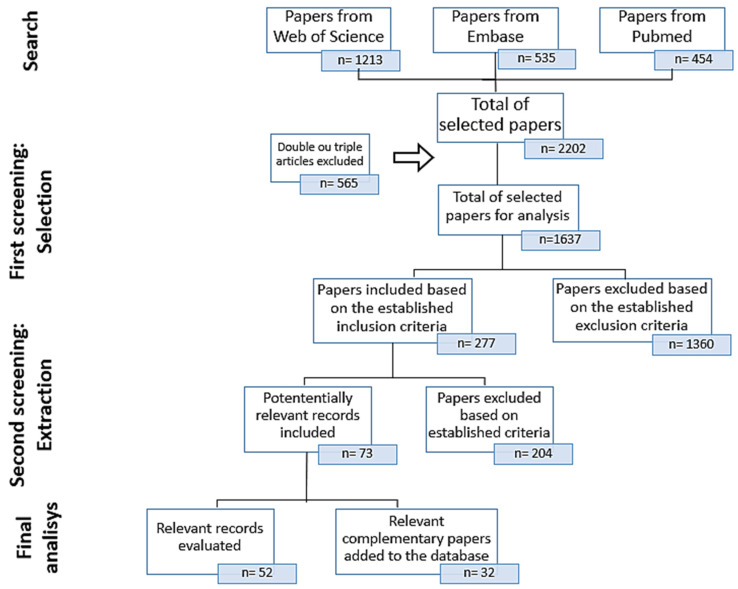
Flowchart indicating the literature search methodology and article selection performed in the present review.

A total of 2202 articles were found, as follows: 1213 in Web of Science, 535 in Embase, and 454 in PubMed. From this total, 565 duplicated assessments were excluded (n = 1637), as well as studies whose aim was the evaluation of matrices containing PAHs, such as crude oil, water, contaminated soil, and sediments or even paper, that were not aligned with our objective (n = 1360). The other 277 papers were fully evaluated in order to disregard studies with missing data or that did not comply with the criteria established for the “Extraction” stage (see reasons in the methodology). Of these, 204 were discarded, leaving 73 studies for analysis. (Figure 3). 

From this, 1 study did not make clear the object of analysis (reagent or environmental sample), and another 12 studies were also excluded because their aim was to test equipment calibration, develop better quantification and identification methods, and/or apply mathematical models for dispersion calculations and risks using secondary data. Subsequently, 32 extra articles containing essential information such as the dynamics and behavior effects of PAHs on aquatic biota, factors that influence toxicity, and data concerning human health risks were added to the database (Figure 3).

**Figure 4:**
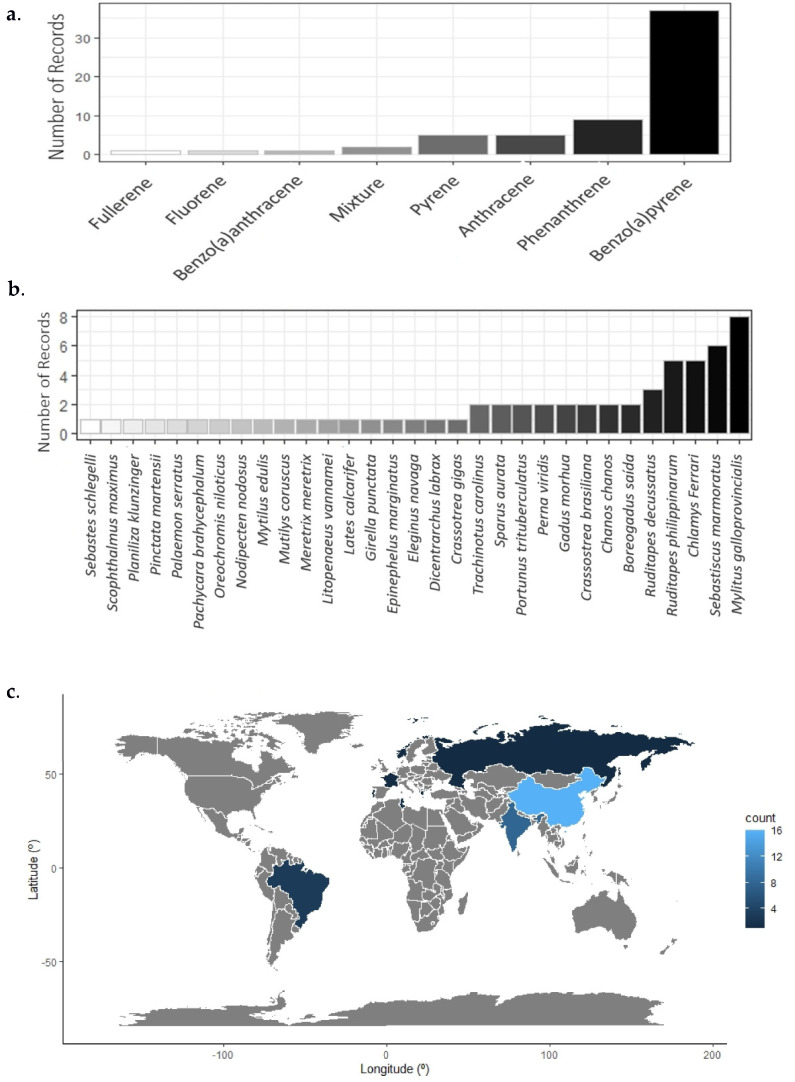
Frequency of assessed PAHs (**a**), species (**b**), and country (**c**) obtained in this systematic review.

From the 52 included articles, we identified only studies concerning 6 of the 16 priority PAHs (pyrene (PYR), phenanthrene (PHE), fluoranthene (FLU), benzo(a)pyrene (BaP), benzo(a)anthracene (BaA), anthracene (ANT)) with fullerene (C60), and two articles registered ecotoxicological effects from tests performed with complex hydrocarbons mixtures employing both the aforementioned PAHs and naphthalene (NAP) and chrysene (CHR) (Figure 4a).

Thirty-one animal species were identified, grouped into five categories: fish, shrimp, bivalves, and crabs/Decapoda, and distributed as animals obtained in field samplings (n = 51) or maintained in the laboratory (n = 3). These assessments were carried out in 19 countries. The bivalve *Mytilus galloprovincialis* was the most studied test organism (Figure 4b), and China published the most studies (n = 32) (Figure 4c).

## 4. Discussion

### 4.1. PAH Assessments

A total of 274 articles were excluded because, although laboratory exposures with standardized test organisms were performed, the test agent was a solution obtained from spill oils or similar (dilbit). In this regard, it is important to note that factors such as compound bioavailability, solubility, volatilization, and half-life can alter PAH toxicity in aquatic media [31], and the chemical composition of crude oil or oil products may not reflect the effect of each individual PAH [32]. 

Phenanthrene was established herein as causing the most significant damage to the survival of both fish and mussels, while anthracene and naphthalene exposure result in sublethal effects, such as damage to genetic structures and delayed or reduced reproduction. Govers et al. (1984) [33] indicated a correlation between the PAH partitioning coefficient (KOW) and toxicity, while Carls and Mador (2009) [34] report that high molecular weight soluble PAHs become more toxic and persistent in nature due to extended half-lives. However, in general, PAHs with lower molecular weights and, thus, fewer aromatic rings (2–3) tend to cause more significant acute toxicity effects. In contrast, PAHs with higher molecular weight and more than five rings tend to generate more chronic effects, with significantly higher carcinogenic potential [35].

In view of these data, it is paramount to define the base toxicity of each PAH in complex mixtures in order to evaluate environmental PAH dispersion, according to chemical, physical, and toxicological characteristics properties [36] (Table 1). This allows for the extrapolation of ecotoxicological results based on the amount of each toxic compound in complex matrixes, such as oil [37,38], by employing mixing models [39].

### 4.2. Volatility

PAH volatility influences the occurrence of these compounds in different environmental compartments, with more volatile substances becoming more representative in the atmosphere due to burning processes. Naphthalene is one of the most volatile PAHs, leading to uncertainties concerning its toxicity in aquatic organisms [41], as it is easily lost by volatilization and sorption processes. However, most PAHs are relatively non-volatile and poorly soluble in the aquatic environment, leading to high chances of incorporation into bottom sediments [42].

**Figure 5:**
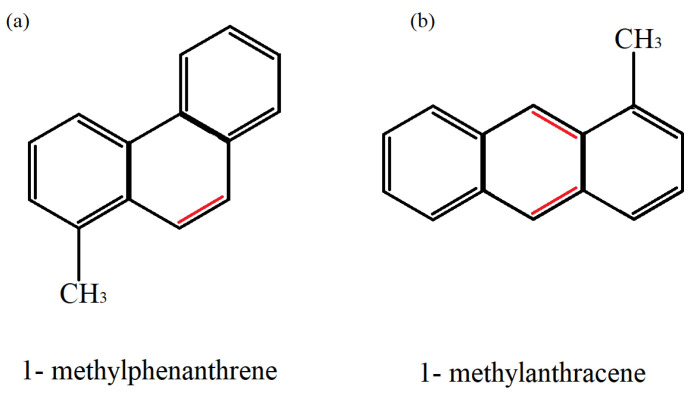
Example of two linear isomers (**a**) 1-methylphenanthrene and (**b**) 1-methylanthracene, with changes in the angular orientation of the molecule and position of double bonds indicated in red.

As indicated in Table 1, low molecular weight PAHs (LMW) also tend to exhibit the lowest KOW, except for isomerism cases, such as the linear isomers 1-methylphenanthrene and 1-methylanthracene (Figure 5). Both exhibit the same structural formula and have one methyl radical (CH_3_) but different angulation and double bond arrangements. For example, while anthracene (ANT) is a linear PAH, phenanthrene (PHE) is the simplest non-linear PAH, and this type of arrangement leads to differences between forces of attraction between the atoms that make up these molecules, making them more or less stable [43].

Despite the generic relationship between molecular weight and volatility, PAHs such as naphthalene may be harder to apply in bioassays due to the loss of these agents by photodegradation adsorption, mainly volatilization [44]. Similar losses have been observed for BaP [14], PHE, ANT [45], and fluoranthene [46]. BaP 500 and 1000 µg L^−1^ can trigger histological and DNA damage in the bivalve *Mytilus galloprovincialis* within 72 h of exposure [47], although concentrations up to 10-fold lower already affect the detoxification system of these bivalves [48]. At 100 µg L^−1^, anthracene and phenanthrene alter both enzymatic and non-enzymatic antioxidants such as AChE and triglycerides [45].

### 4.3. Molecular Weight Influence

PAHs are often categorized according to the number of aromatic rings as high molecular weight (HMW, 4–6 rings) and low molecular weight (LMW, 2–3 rings) compounds [49]. HMW compounds are usually more associated with genotoxicity because of their higher chronic effects [50]. This occurs due to their degradation processes, as HMW PAHs are more persistent, directly correlated to fugacity and vaporization rates due to their higher KOW [31].

The effects of acute and long-term exposure to PAHs in marine animals as a function of their molecular mass are commonly generalized between severe physiological damage and mortality for LMW compounds and cancer, although [51] indicated that PAHs do not act as direct carcinogens, with their metabolites being, instead, responsible for this deleterious process. Thus, HMW PAH genotoxicity is, in fact, caused by their biotransformation processes. According to Baird et al. (2005) [52], cancer associated with PAHs is due to the effects of products of the reaction of the K-region epoxide of these compounds, such as 7-methylbenz[a]anthracene (7-MeBa) and BaP with DNA, although the form of these products may vary depending on the assessed PAH. In fish, for example, both LMW and HMW compounds are routinely detected, although LMW compounds are generally present in higher concentrations [53] as these animals easily metabolize these compounds compared to other organisms such as mollusks [54]. Assessments concerning PAH-associated genotoxicity in animals must, therefore, consider that following effective exposure, i.e., contact with assimilable PAH forms; these compounds undergo activation in animal metabolism by oxidative processes (epoxide formation) that promote the formation of adducts between these substances and DNA, leading to mutations [52].

The retrieved papers in our systematic search indicate that anthracene (ANT) is a common contaminant in estuaries and coastal areas, having been reported at concentrations of up to 35 µg L^−1^, despite being reported as not resulting in lethal effects in fish *T. carolinus* (Florida pompano) at this concentration. However, this estuarine fish feeds on molluscs, crustaceans, and other small fish [55], potentially bioaccumulating this PAH, indicating that even as an LMW compound, ANT can affect organisms at environmentally realistic concentrations. This PAH has also been associated with increased enzymatic activity, such as catalase (CAT), and protein concentrations, such as glutathione (GSH) in *Chanos chanos* (milkfish) tissues [56]. This fish typically inhabits the coastal zone and may even enter streams due to a diversified diet, ranging from phytoplankton to invertebrates and small fish. Furthermore, ANT has been reported as lethal for milkfish at 0.30 mg L^−1^ after 96 h of exposure [56], 8.57-fold higher than that for *T. carolinus* [55], demonstrating different tolerances between species and test concentrations, which can be explained by their different trophic chain positions.

In turn, phenanthrene (PHE), also an LMW PAH; it can linearly affect the cardiovascular system of Navaga cod fish (*Eleginus navaga*) through changes in cellular ion fluxes, resulting in cardiac dysfunction at doses ranging between 1 and 30 µmol L^−1^ [57], also leading to hormetic spermatogenesis responses in the fish *Sebastiscus marmoratus* (sea ruffe) [11], with lower PHE concentrations inhibiting the development of testes, while a higher concentration (6 µg L^−1^) was less inhibitory concerning testicular development.

The fish *S. maroratus* (sea ruffe) is also sensitive to BaP, PYR, and PHE at environmentally relevant concentrations, undergoing expressive mortality at 0.1 µg L PHE exposure after eight days in tanks with water and PAH. Although BaP is an HMW compound and the most addressed PAH in the studies included in this systematic review, mortality was not indicated as the primary endpoint for this substance in this species, even the following exposure at 1 µg L^−1^, with 10% of the exposed animals dying, while for PHE, this same percentage was exceeded in a 10-fold lower concentration, although BaP affects certain endpoints, such as craniofacial skeletal development, more than PHE [58]. Although these facts are not explained based on eventual differences of these two PAH mechanisms, this indicates that molecular mass alone cannot define PAH lethality, although the mechanism for these effects was not investigated in the retrieved assessments.

The importance of assessing primary toxicity endpoints, such as histological and biochemical changes, as a response to PAH toxicity becomes clear as lethality is not always observed.

### 4.4. Lipophilicity

Lipophilicity is one of the main physicochemical PAH properties that result in seafood contamination [46,59], as lipid affinity makes PAHs likely to interact with lipid contents, such as animal cell membranes. For example, Honda et al. [60] point out that increased lipid content in marine fish may indicate the specific accumulation of PAH in particular tissues, altering the lipid metabolism, resulting in endoplasmic reticulum dysfunctions, among others.

Concerning benzo(a)pyrene (HMW PAH), several effects of this PAH on DNA have been reported, although the selected articles in our systematic review assessed mainly cytological and histological alterations as well as Phase I biotransformation biomarkers. Concerning anthracene, an LMW PAH, two studies evaluated fish (*Trachinotus carolinus*—Florada pompano and *Chanos chanos*—milkfish) and one, the Mediterranean mussel *M. galloprovincialis*. In the fish assessments, DNA alterations were verified employing the Comet assay [55], and survival rates were assessed [56], while oxidative stress biomarkers and filtration rates were the evaluated endpoints concerning the bivalve study [44,61].

In a comparative analysis for these two PAH, Palanikumar et al. [56] reported that *C. chanos* exposed to anthracene and benzo(a)pyrene (BaP) exhibited a directly proportional increase in accumulation levels as a function of PAH concentrations. Furthermore, BaP was found to be more toxic to *C. chanos* compared to anthracene, confirming the linearity between molecular mass, lipophilicity, and toxicity, as even at 3-fold higher concentrations, ANT exposure resulted in minor survival effects, acetylcholinesterase activity alterations, and Phase 2 detoxification enzymes compared to the higher mass compound [50,61].

### 4.5. Environmental Chemistry and PAH Dispersion

Environmental factors can significantly alter PAH toxicity [62]. Although heat and light incidence can increase their degradation [63], higher temperatures favor increased toxicity of certain PAHs such as phenanthrene, naphthalene, and anthracene due to increased kinetics, accelerating intoxication processes [64]. Moonfish exposed to anthracene, for example, exhibited increased mortality due to higher water temperatures and oxygen levels [65].

Furthermore, environmental characteristics such as geomorphology, environment, and ecology also significantly influence ecotoxicological endpoints. For example, in Gulf killifish, increased mortality and development alterations in the larval stage were observed as a function of temperature and hypoxia, with both variables highlighted as potential detoxification pathway inhibitors [66]. Climatic conditions, solar incidence, and seasonal changes can also generate toxicity differences concerning xenobiotic dispersion in aquatic media [28].

HMW PAHs are more abundant in the atmosphere and high-temperature environments due to combustion processes [50]. High temperatures also favor sedimentary particle sorption and desorption as well as toxicity [67]. Furthermore, environmental temperatures can influence individual tolerance through physiological strategies developed for acclimatization to extreme temperature conditions, such as extreme cold. Arctic cod, for example, present low gastric evacuation rates and high lipid assimilation efficiency, which favor PAH tissue distribution [68].

**Figure 6:**
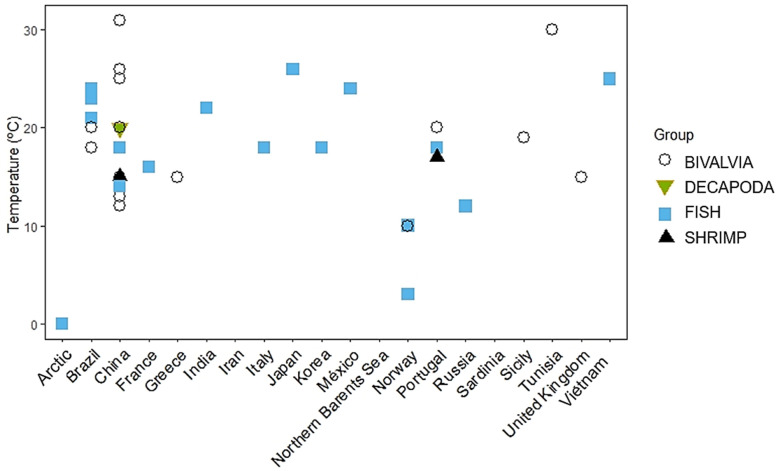
Different taxonomic groups by region/country and applied temperature ranges in the studies evaluated in our systematic review.

Figure 6 displays the different taxonomic groups evaluated by region/country and applied temperature ranges in the studies retrieved in this systematic review.

Some differences between response patterns were observed concerning the wide PAH concentration ranges applied at different temperatures (Figure 5). In studies conducted in China, for example, *Ruditapes philippinarum*—Manila clam—(n = 5 studies) was subjected to a range of 12 to 25 °C, while *Chlamys farreri* (n = 4 studies) underwent bioassays from 15 to 31 °C. (Figure 5). Despite the high amplitude for this condition, only one study recorded significant temperature effects on PAH toxicity [68].

The bivalve *Ruditapes decussatus* can respond differently to the same chemical compounds (FLU, PHE, and PYR) at different temperatures (20, 24, 28, and 30 °C), even altering their defense mechanisms [69]. A significant effect on total hemocyte counts was observed only for FLU at different temperatures and concentrations, while PYR at 20 °C also increased this parameter, confirming that PAH effects depend on both the evaluated PAH and temperature.

PAH effects may also vary with oxygen and salinity variations [66,70], with decreased salinity values increasing toxic PAH effects [70]. Several studies indicate that salinity fluctuations promote a need for physiological compensation, such as gill activity. These structures are responsible for ion regulation and comprise the first barrier against xenobiotics, and salinity variations can result in PAH damage to the gill epithelium.

Despite variations in temperature, salinity was constant in the selected studies, ranging between 30% and 37.5% without restriction regarding species or location. Only two papers differed, one establishing a salinity value of 20% when assessing blue mussel *M. edulis* [46] and another evaluating the effect of increased salinity values on the *Crassostrea brasiliana* transcriptome, which reported that PHE exhibited decreasing levels in tanks containing only water, even without animals, probably due to its low solubility in saline solutions [49].

In turn, oxygen was determined in 6 of the 32 analyzed articles, ranging from 6.5 to 8.6 mg L^−1^ [43,45,56,59,71,72,73]. Besides its possible participation in PAH transformation processes, oxygen plays a vital role in osmoregulation, and its consumption can be interrupted by toxicant action in gill tissues, affecting osmoregulation [43].

### 4.6. PAH Toxicity in Mixtures

Laboratory exposures of aquatic organisms to combined contaminants (xenobiotic cocktails) and adsorbed to other materials, such as plastic or carbon nanotubes and microplastics, may lead to additive, synergistic or antagonistic effects [48], indicating environmentally realistic situations [74,75], sometimes at concentrations below individual contaminant lowest observed effect concentration (LOEC) [76]. For example, PAH absorbed by these materials became bioavailable and toxic to the algae *Pseukichneriella subcapitata* [77]).

As indicated in Table 2, nine of the selected articles exposed PAHs as mixtures to marine organisms. Of these, four involved PAH coexposures, two were based on binary mixtures and two on complex mixtures, in addition to studies conducted employing metals (copper—Cu), nanoparticles (TiO_2_NP), microplastics (MPs), and organic pesticides (i.e., dichloro-diphenyl-trichloroethane, DDT).

Interactions between BaP and fullerene (C60) generated antagonistic effects concerning genotoxic and proteome expressions, significantly increasing DNA strand breaks following three days of exposure to 0.1 mg L of a mixture of both compounds when compared to the control and individual treatments [48]. On the other hand, individual and combined PHE and ANT treatments led to total thiol status alterations, which may result in physiological and morphological mussel gill alterations [45]. The observed antagonistic effects between BaP and C60 cannot be explained by B[a]P sorption onto C60 but rather by the free radical scavenging property of C60, as single and combined exposures resulted in common response mechanisms of transcriptomic alterations related to genotoxic mechanisms [48]. Concerning the mixture between PHE and ANT, the authors indicate that the absence of observed additive effects may be due to exposure adaptation during the 7-day exposure period.

Song et al. [80] recorded changes in the levels of some metabolites associated with the single exposure to 10 µg L^−1^ BaP in *Perna viridis* mussel gills, with some amino acids from energy metabolism, such as BCAAs, dimethylamine, and dimethylglycine, significantly reduced while proteins involved in cytoskeleton organization, catabolic protein, and apoptosis were increased. However, no metabolic changes in a 1:1 mixture with DDT, a pesticide of global concern due to its high persistence in environmental compartments, were observed, suggesting antagonistic effects between BaP and DDT that may be linked to their different metabolic pathways [80].

However, for exposure to BaP + C60, ANT + PHE, and BaP + DDT mixtures, the concentration of the second toxicants was set as a constant, modifying only the investigated PAH concentrations. This experimental design is relevant for understanding the interaction between substances but can also be restrictive and non-environmentally relevant. Thus, non-additive and antagonistic interactions may occur at different doses intervals than those tested in the laboratory. For example, different sets of proteins and complementary modes of action were observed when analyzing mussel gills exposed to BaP, Cu, and their mixture [62]. Unlike other studies, no BaP accumulation was observed, which may be associated with competitiveness between the tested compounds and consequent greater metal absorption, although the interaction between PAH and Cu leads to common response mechanisms.

### 4.7. Factors That Can Affect the Toxicological Response of Animals to PAH

Laboratory tests aim to verify contaminant effects on test organisms in a controlled manner. However, the response may vary between different taxonomic groups due to varying tolerances and a significant variety of laboratory protocols. Thus, this topic will address the main differences in conducting ecotoxicity bioassays with PAHs in the papers selected in this systematic review.

Two studies in our systematic review [46,73] (Table 2) aimed to evaluate the effect of combined exposure to PAHs and microplastics. Pyrene (PYR) is commonly associated with adverse outcomes in fish, but when associated with microplastics (MPs), sublethal damage or no effects have been reported in studies when each compound was tested individually [73]. However, the mixture between these agents quickly affected juvenile barramundi (*Lates calcarifer*) predatory performance [73]. In turn, single fluoranthene exposure resulted in bioaccumulation in both the gills and digestive gland of the blue mussel (*Mytilus eduli*), which was not observed when adding MPs [46], even though MPs are considered an important vector for many pollutants.

Interestingly, mussels previously kept in clean water subjected to FLU and MP coexposure bioaccumulated more FLU than animals subjected to a single FLU exposure. However, despite the clear hypothesis of the additional effect of PAH adsorption to particles, an mRNA analysis suggested that the presence of MPs alters detoxification activity [46]. This information is vital for understanding multiple-effect pathways since it is suggested that extra-biological interaction, i.e., a crude mixture between toxic agents, is the main pathway of cointoxication. Thus, as observed between DDT and BaP [80], different xenobiotic binding sites can generate complimentary or overlapping biological response pathways.

Furthermore, the potential for adsorption onto surfaces and particles can make it difficult to accurately assess effects under controlled conditions. The tendency of BaP to adsorb to aquaria walls, for example, may explain changes in PAH concentrations in exposure bioassays, although the composition of these tanks (plastic or glass) was not indicated in the studies investigated herein [81]. Different solvents and tank compositions should, therefore, be tested. For example, the use of acetone as a solvent for BaP in some tanks may favor BaP losses through evaporation and adsorption, which seems to be associated with a high abrasive potential that can also remove lipids and proteins [81].

### 4.8. Aquatic Biota PAH Exposure Studies

The ecotoxicological studies obtained in our systematic review (Table 3) mostly focused on the evaluation of effects on aquatic invertebrates, such as zooplankton and small fish. However, bivalves like the blue mussel were also noteworthy and frequently employed as a model species due to their wide geographic distribution and filter-feeding characteristics, making them susceptible to bioaccumulation processes, thus demonstrating their adequacy as PAH contamination bioindicators or sentinel species [82]. It is also important to note the important bivalve role as a significant food source worldwide. For example, from 2009 to 2018, global mussel aquaculture production increased by 8%, driven mainly by the growth of the Spanish output. In 2018, for example, the EU provided 84% of the global production of *Mytilus galloprovincialis*, represented by both live and fresh mussels (44%) and prepared and preserved mussels (39%) [83].

The study performed by Speciale et al. (2018) [47] best describes the One Health concepts mentioned previously, associating animal and human health risk endpoints. The authors report that the acute exposure of blue mussels to BaP is capable of causing pathological changes in gills, which confirms the biotransformation activity of this tissue due to PAH intoxication. In addition, PAH CYP1A bioactivation is associated with DNA damage and carcinogenic potential [90]. Thus, CYP1A may comprise a valuable tool as a human health risk assessment biomarker. Furthermore, exposure of mononuclear cells to contaminated products by BaP at levels similar to human ingestion rates demonstrated toxic potential with morphological alteration to mussels at 0.5 mg L^−1^, which effectively indicates that the consumption of contaminated mollusks constitutes a significant risk to human health [47].

### 4.9. Ecotoxicological Responses

Even though environmental chemistry can assess PAH effects under different conditions, ecotoxicology establishes the flow of these contaminants in the biota and the effects on individuals’ interaction populations. Furthermore, this field is essential for understanding environmental and ecological scenarios and evaluating the effects of chemical and xenobiotic substances on food chains as well as being economically relevant [108], as these data contribute to public health maintenance through risk assessments.

Several trials selected in our systematic review evaluated the deleterious effects of exposures in different media (sediment and/or water), simulating what naturally occurs in the aquatic environment. For mollusks and crabs, exposure to a stock solution of the test PAH in tanks/aquaria containing water and sediment was the most commonly applied method, followed by exposure to PHE, BaP, FLU, ANT, or BaA for intervals ranging from 24 h to 50 days (Table 3).

In one of the assessments, the BaP did not significantly bioaccumulate in exposed Klunzinger’s mullet tissues, despite intraperitoneal injection applications increasing the activity of superoxide dismutase (SOD) and cytochrome P450 enzymes [83]. In turn, when subjected to this PAH, bivalves not only accumulated this compound in tissues, especially gills, but also suffer reproductive organ damage, i.e., ovarian development inhibition and damage to ovarian envelope connective tissues [86], indicating the magnitude of different responses observed in this type of assessment.

### 4.10. Biomarker Evaluations

Only two studies efficiently addressed the relationship between ecotoxicology and potential human health risks [47,59], while the other 51 selected studies and the extra references added to this study contributed mainly to parameter definitions and endpoints from the intoxication by different PAHs in animals that constitute the food base of many human populations. As discussed throughout this document, the two aforementioned studies evaluated the effects of BaP in marine animals (blue mussel and sea bream, respectively) and possible implications for human health. While [47] exposed mollusks to 1 mg L^−1^ of pyrene 72 h, [59] subjected the marine fish *Sparus aurata* to double this amount (2 mg L^−1^) for the same period and time, also exposing human peripheral blood mononuclear cells. The findings obtained in these assessments may, therefore, be useful in indicating common enzyme markers for both animals and humans, which may, in turn, indicate an evaluation route for concerning human exposure due to contaminated seafood diets.

**Figure 7:**
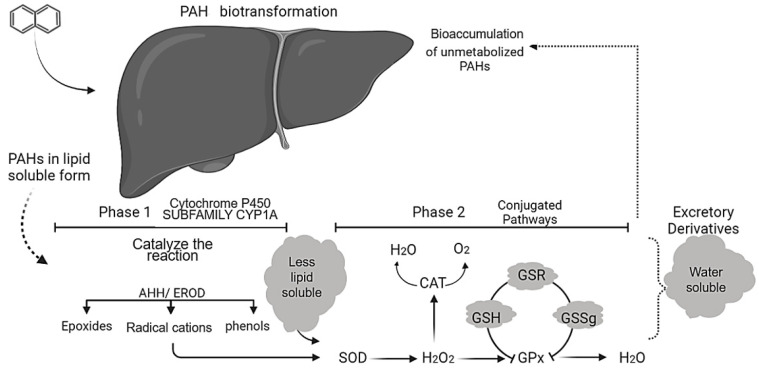
PAH biotransformation pathway scheme indicating intoxication steps, CYP1A-mediated biotransformation phase I, antioxidation enzymes that act against ROS during Phase II, and water-soluble conjugate excretion. AHH—aryl hydrocarbon hydroxylase; EROD—ethoxyresorufin O-deethylas; SOD—superoxide dismutases; CAT—catalase; GPx—glutathione peroxidase; GSH—reduced glutathione; GSR—glutathione reductase; GSSg—glutathione disulfide.

However, a common mechanism of action has not yet been well-explored, even though PAH effects on Phase 1 and 2 biotransformation detoxification markers are clear (Figure 6). This process, essential in xenobiotic detoxification, is initiated by the transcription of enzymes by the aryl hydrocarbon receptor (AhR) pathway, which can also be applied as a PAH toxicity biomarker [92]. The enzymes involved in this process, such as the cytochrome P450 enzymes that act in Phase 1, can be classified as a function of the biotransformation stage during which they act, with polarity being responsible for transforming liposoluble compounds into more water-soluble forms, easier to excrete [86], typically generating different metabolites. Some enzymes also act in the conjugation of these metabolites (Phase II), resulting in xenobiotic conjugates, which are more readily excreted (Figure 7) [59,92].

The CYP1A subfamily (Figure 7) is the most studied among the cytochrome P450 family of enzymes as it is significantly induced by PAHs. The activity of this marker in fish and shellfish has been applied in many assessments to verify PAH effects, especially BaP, which significantly increases the expression of CYP1A as a function of exposure time and dose [47,59,85,86,87,88,89,92,93,100].

Despite CYP1A mediation, metabolite formation can be harmful to many organisms, even though these products can also be applied to assess intoxication. Thus, the metabolic mechanism of PAHs is also an essential point of attention for the ecotoxicological evaluation of these compounds. For example, concerning Phase I enzymes, the oxidation process of BaP to epoxides and phenols has a better description. Following this phase, epoxides undergo one to two hydrolysis by epoxide hydrolase and, after second CYP-mediated oxidation, are converted into di-olepoxides with high carcinogenic potential [80] Furthermore, metabolites such as di-hydrodiols B can be oxidized to quinones by di-hydrodiol dehydrogenase [96]. This process commonly leads to reactive oxygen species (ROS) formation, which is detoxified by the protective antioxidant system. This system comprises several enzymes and proteins, such as superoxide dismutase (SOD), catalase (CAT) and reduced glutathione (GSH), glutathione-S-transferase (GST), and glutathione peroxidase (GPx), which accumulate in important protective metabolic pathways and serve as oxidative stress biomarkers [56,80].

GSH, a non-enzymatic biomarker, has been the target of several PAH exposure assessments, including FLU, ANT, PHE, and BaP [45,53,55,82,96]. Decreased GSH levels were reported for gills in *M. edulis* subjected to FLU individually and combined with microplastics [46] and in *M. galloprovincialis* exposed to single treatments and a mixture of ANT and PHE, while increased GSH levels were observed in milkfish following anthracene exposure. On the other hand, decreased GSH levels were reported following BaP exposure in milkfish [56] which reinforces the different responses between species and, above all, the effects of different doses and exposure durations (Table 3).

In addition to biotransformation responses, which comprise quick response biochemical pathways, PAH can also alter morphological and physiological components in addition to impairing survival. PHE, for example, is known for its potential to induce DNA damage and disturb aquatic organism behavior in addition to affecting the hepatocyte area and resulting in lethality for certain fish species, such as *Epinephelus marginatus* (LC_50_ 1.51 mg L^−1^) [71]. ANT has been shown to compromise the swimming behavior of *Palaemon serratus* fish by reducing swimming speeds at environmentally relevant concentrations from 128 ug L^−1^ [72,109] (Table 3). From 150 nmol L^−1^ PYR, the fish *Lates calcarifer* exhibited increased immobility and decreased survival rates to decreased feeding rates [73] (Table 3).

Thus, in addition to direct animal health effects, PAHs, even though distinct in terms of chemical structures, environmental dispersion, and metabolism pathways, can alter ecosystem dynamics through damage to key species and even indirect damage to trophic interactions [14]. Therefore, ecotoxicological studies are paramount to determining PAH levels resulting from human–environment interactions, establishing no-effect values that also indicate no risks to human health [59].

### 4.11. The Relationship between Toxic Limits and Different Risk Concepts

The consumption of contaminated fish is a potential source of risk, increasing the chances of several deleterious effects in humans [110]. Risk assessment articles usually focus on human effects and do not evaluate environmental impacts, while ecotoxicological studies, even if carried out in controlled environments, tend to conclude that their findings constitute a basis for developing quality control standards for public management. However, it is important to note that joint efforts in both areas should be carried out as a theoretical basis can contribute to decision-making aiming at decreasing aquatic contamination effects and improving human and environmental quality.

In our systematic review, only papers assessing the interface between toxicity values and risk of exposure to biological matrices able to trigger disorders in human beings were assessed, while articles associated with mathematical models from meta-analyses or development studies regarding purely risk calculation methodologies or risk models were excluded. This led to the selection of only two articles that evaluated the link between ecotoxicological methods and human health effects, although with no risk assessment modeling efforts carried out.

According to the model developed by the US Environmental Protection Agency [111], direct particle ingestion, inhalation, and dermal contact as exposure routes should be considered. For non-carcinogenic compounds, the risk is estimated using a hazard index (*HI*), which is equal to the sum of hazard quotients, calculated as *HI* = *HQ_ing_* + *HQ_inh_* + *HQ_dermal_*, as follows:

(1) $$HQ_{ing}=C\times \frac{lnR\times EF\times ED}{BW\times AT}\times 10^{-6}$$

(2) $$HQ_{inal}=C\times \frac{lnhR\times EF\times ED}{PEF\times BW\times AT}$$

(3) $$HQ_{dermal}=C\times \frac{SL\times SA\times \left|\times \right|EF\times ED}{BW\times AT}\times 10^{-6}$$

where *HQ_ing_* corresponds to the toxicant ingestion (mg kg^−1^ day^−1^), *HQ_inal_* refers to inhalation, *HQ_dermal_* is the dose associated with dermal contact, *C* is the concentration of the contaminant agent in the exposure matrix, in mg kg^−1^. InhR is the mean ingestion rate of the contaminated matrix; while *EF*, *ED*, *BW*, and *AT* are the exposure frequencies (180 days year^−1^), duration of exposure (years of consumption), average body weight (15.0 kg^3^ for children and 70 kg for adults), and average time (*DE* × 365 days). These equations can also be simplified as:

(4) $$HQ=\frac{D}{RfD}$$

where *HQ* is the hazard quotient obtained by the ratio between the dose of the contaminant in mg kg^−1^ (*D*) and its reference dose (*RfD*). *HQ* values are obtained through the quotient between the maximum concentrations of the studied substance, and the predicted no-effect concentration (PNEC), considering the effect concentration (EC_50_), lethal concentration (LC_50_), or even the non-observed effect concentration (NOEC), which are usually available in the literature or can be discovered employing bioassays, further linking the ecotoxicology and human health risk assessment fields.

A fundamental difference is noted, however, between theoretical and modeled risks. While the theory involves calculating the probability of effect from the ratio between potential exposures and effect concentrations [112], in practice, the risk quotient is obtained from predictive models, which uses the bases of toxicology, also applied to define legislation levels, in comparison to predicted environmental concentrations [113].

In turn, ecological risk assessments aim to characterize the probability of occurrence of environmental effects resulting from human actions. This field of research favors effect assessments on organisms (animals and plants) that make it possible to verify xenobiotic ecological toxicity. This index can be estimated by comparing the studied substance’s hazard quotient (*HQ*) with its corresponding environmental quality value. However, as the lack of data on the individual toxicities of PAH can be a challenge in this regard, some researchers have agreed that a PAH toxicity equivalence factor (TEF) can be used in blank dates due to similar ecological and human health effects [114].

(5) $$CQ=\frac{C_{m}}{C_{qv}}$$

where *CQ* is the risk quotient provided by the ratio between *C_m_*_,_ the PAH concentration in the studied matrix (e.g., water), and *C_qv_*, the quality value that considers the permissible concentrations.

Environmental risk assessment is, therefore, essential to determine whether pollutants present in water bodies threaten aquatic biota and human beings. Thus, concentrations in the marine environment and their toxicity data concerning different organisms become paramount in determining the risks of these compounds.

## 5. Conclusions

Even with the increasing number of studies aimed at the applicability of environmental and public health concepts in conjunction with the One Health concept and the urgent need for information to aid in the determination of safe levels of toxic agents, laboratory tests and field studies still appear to be the greatest source of data for conducting human health risk assessments.

Temperature, dissolved oxygen, and salinity fluctuations, as well as intrinsic physicochemical properties, can affect PAH availability and toxicity. Their interactions with each other and with other contaminants of anthropic origin are also of note, with different effects on marine organisms that could, possibly, affect human health. This should be further addressed by ecotoxicology assessments.

Tests on commercially important organisms such as bivalve mollusks, crabs, and fish tend to compose most risk assessments. Studies concerning fishery products have, in fact, increasingly evaluated several PAH intoxication markers as putative indicators for animal health problems, despite a significant lack of investigations concerning the potential associations to human health effects being noted. However, our systematic review still makes it clear that environmental and ecological aspects are still mainly studied separately, demonstrating that multidisciplinary assessments regarding PAH toxicities are urgently required. This can be evidenced by the fact that only 2 studies among the 1360 selected studies make the connection between animal and human health in a connected way, highlighting a gap in knowledge.

## Figures and Tables

**Table 1:** Physical and chemical PAH properties that influence environmental PAH dispersion. Source (Pubchem, 2012) [40].

Name	Structural Formula	Molecular Weight	Partition Coefficient (Log KOW)	Vapour Pressure (25 °C)
Anthracene	C_13_H_8_	180.20	3.58	5.7 × 10^−5^
Fluorene	C_13_H_10_	166.22	4.18	6.00 × 10^−4^
Phenanthrene	C_14_H_10_	178.23	4.46	1.21 × 10^−4^
Methylphenanthrene	C_15_H_12_	192.25	4.97	1.50 × 10^−5^
Methylanthracene	C_15_H_12_	192.25	-	5.34 × 10^−6^
Pyrene	C_16_H_10_	202.25	4.88	4.50 × 10^−6^
Fluoranthene	C_16_H_10_	202.25	5.16	9.22 × 10^−6^
Benzo(a)antracene	C_18_H_12_	228.3	5.76	2.1 × 10^−7^
Crysene	C_18_H_12_	228.3	5.73	6.23 × 10^−9^
Benzo(b)fluoranthene	C_20_H_12_	252.3	5.78 (0.0015 mg L^−1^)	5.00 × 10^−7^
Benzo(k)fluoranthene	C_20_H_12_	252.3	6.11	9.65 × 10^−10^
Benzo(e)pyrene	C_20_H_12_	252.3	6.44	5.70 × 10^−9^
Benzo(a)pyrene	C_20_H_12_	252.3	6.13	5.49 × 10^−9^
Indeno[1,2,3-cd]pyrene	C_22_H_12_	276.3	6.70	1.3 × 10^−10^
Dibenz(a,h)anthracene	C_22_H_14_	278.3	6.50	9.55 × 10^−10^
Benzo(g,h,i)perilene	C_22_H_12_	276.3	6.63 (9.41 mg L^−1^) *	1.0 × 10^−10^
Coronene	C_24_H_12_	300.4	-	2.17 × 10^−12^

* Solubility in water at 25 °C.

**Table 2:** Binary and complex PAH exposure mixtures reported in the selected articles in this systematic review.

Species	Common Name	Compound	PAH Concentrations in Single Treatments	Compound Concentrations in Single Treatments	Total Mixture Concentrations	Reference
*Mytilus galloprovincialis*	Mediterranean Mussel	PHE ANT	100 µg L^−1^ 100 µg L^−1^	-	100 µg L^−1^ (50 µg L^−1^ each)	[45]
*Gadus morhua*	Atlantic cod	NAP, PHE, dibenzothiophene (DBT), PYR BaP, FLU	-	-	12.64 µg kg^−1^ 8.38 µg kg^−1^ 0.58 µg kg^−1^ 1.45 µg kg^−1^ 1.93 µg kg^−1^ 15.03 µg kg^−1^	[78]
*Scophthalmus Maximus*	Turbot	NAP, ANT, PHE, FLU, PYR, CHR, BaP	-	-	10,600 mg L^−1^ 10,200 mg L^−1^ 7500 mg L^−1^ 13,300 mg L^−1^ 3300 mg L^−1^ 15,500 mg L^−1^ 5200 mg L^−1^	[44]
*Mytilus galloprovincialis*	Mediterranean Mussel	BaP C60	5, 50, and 100 µg L^−1^ of BaP	10, 100, and 1000 µg L^−1^ of C60	1000 µg L^−1^ of C60 + 5 µg L^−1^ of BaP 1000 µg L^−1^ of C60 + 50 µg L^−1^ of BaP 1000 µg L^−1^ of C60 +100 µg L^−1^ of BaP	[48]
*Mytilus galloprovincialis*	Mediterranean Mussel	BaP (Cu)	10 µg L^−1^ of BaP	10 µg L^−1^ of Cu	10 µg L^−1^ of BaP + 10 µg L^−1^ of Cu	[62]
*Lates calcarifer*	Barramundi	PYR MPs	100 nM of PYR	100 MP L^−1^	100 nM of PYR + 100 particles L^−1^	[73]
*Mytilus edulis*	Blue mussel	FLU MPs	50, 10 µg L^−1^ of FLU	100, 1000 MP mL^−1^	100 µg L^−1^ of FLU + 1000 MP mL^−1^ 50 µg L^−1^ of FLU + 100 MP mL^−1^	[46]
*Mytilus edulis*	Blue mussel	BaP TiO_2_NP	20 µg L^−1^ of BaP	0.2, 2 mg L of TiO_2_	20 µg L^−1^ BaP + 0.2 mg L^−1^ TiO_2_NP 20 µg L^−1^ BaP + 2 mg L^−1^ TiO_2_NP	[79]
*Perna viridis*	Green mussel	BaP DDT	10 µg L^−1^ of BaP	10 µg L^−1^ of DDT	20 µg L^−1^ (10 µg L^−1^ of each one)	[80]

**Table 3:** Employed test species and their respective effect concentrations for the endpoints evaluated in exposures to different PAHs obtained in our systematic review.

Species	Common Name	Reference	Compound	Concentrations	Exposure Time
*Trachinotus carolinus*	Florida pompano	[55]	Anthracene	8–32 µg L^−1^	24 h *
*Girella punctata*	Largescale blackfish	[84]	Benzo(a)anthracene	1 and 10 ng/d dose	10 days
*Chanos chanos*	Milkfish	[56]	Benzo(a)pyrene	0.002–0.031 mg L^−1^	96 h
*Chlamys farreri*	Farrer’s scallop	[85,86,87]	Benzo(a)pyrene	0.025–10 µg L^−1^	10 days
*Crassostrea gigas*	Pacifi cupped oyster	[88]	Benzo(a)pyrene	0.2–5 µg L^−1^	15 days
*Dicentrarchus labrax*	Sea bass	[72]	Benzo(a)pyrene	2–256 µg L^−1^	96 h
*Gadus morhua*	Common cod	[89]	Benzo(a)pyrene	2.52–252.3 µg L^−1^	48 h
*Litopenaeus vannamei* and *Mytilus coruscus*	White shrimp and Korean mussel	[90]	Benzo(a)pyrene	0.03–3 µg L^−1^	21 days
*Mytilus galloprovincialis*	Mediterranean mussel	[48]	Benzo(a)pyrene	5–100 µg L^−1^	3 days
*Mytilus galloprovincialis*	Mediterranean mussel	[47]	Benzo(a)pyrene	0.5 and 1 mg L^−1^	72 h
*Oreochromis niloticus*	Tilapia	[91]	Benzo(a)pyrene	20 mg kg^−1^	120 h
*Pachycara brachycephalum*	-	[92]	Benzo(a)pyrene	10 and 100 mg L^−1^	10 days
*Perna viridis* and *Pinctada martensii*	Brown mussel and Japanese Pearl-oyster	[81]	Benzo(a)pyrene	2–16 µg L^−1^	14 days *
*Planiliza klunzinger*	Klunzinger’s mullet	[93]	Benzo(a)pyrene	5–50 mg kg^−1^	14 days
*Portunus trituberculatus*	gazami crab	[94,95]	Benzo(a)pyrene	0.1–2.5 µg L^−1^	10 days
*Ruditapes philippinarum*	Manila clam	[96]	Benzo(a)pyrene	0.03–3 µg L^−1^	21 days
*Ruditapes philippinarum*	Manila clam	[97,98]	Benzo(a)pyrene	4 µg L^−1^	5 and 15 days
*Ruditapes philippinarum*	Manila clam	[99]	Benzo(a)pyrene	0.02 and 0.2 µmol L^−1^	96 h
*Sebastes schlegelii*	Korean rockfish	[100]	Benzo(a)pyrene	2–200 µg g bw^−1^	48 h
*Sebastiscus marmoratus*	Sea ruffle	[58]	Benzo(a)pyrene	0.01–1 µg L^−1^	6 days
*Sparus aurata*	Gilt-head	[59]	Benzo(a)pyrene	2 mg L^−1^	72 h
*Sparus aurata*	Gilt-head	[101]	Benzo(a)pyrene	10^−4^ to 10^6^ µg L^−1^	72 h
*Trachinotus carolinus*	Florida pompano	[102]	Benzo(a)pyrene	1–8 mg L^−1^	10 days
*Crassostrea brasiliana*	Mangrove oyster	[43]	Phenanthrene	100 µg L^−1^	96 h
*Chlamys farreri*	Farrer’s scallop	[103]	Benzo(a)pyrene	1–8 mg L^−1^	10 days
*Chlamys farreri*	Farrer’s scallop	[104]	Benzo(a)pyrene	1–8 mg L^−1^	29 days
*Meretrix meretrix*	Asiatic hard clam	[14]	Benzo(a)pyrene	1–8 mg L^−1^	24 h
*Mytilus edulis*	Blue mussel	[46]	Fluoranthene	50 and 100 µg L^−1^	96 h
*Ruditapes decussatus*	Carpet shell	[69]	Fluorene	0.1–1 mg L^−1^	24 h
*Boreogadus saida*	Polar cod	[80]	Benzo(a)pyrene	0.1 and 480 µg L^−1^	14 days
*Crassostrea brasiliana*	Mangrove oyster	[105]	Phenanthrene	100 and 1000 µg L^−1^	24 h
*Eleginus navaga*	Atlantic navaga	[57]	Phenanthrene	1–30 µmol L^−1^	-
*Epinephelus marginatus*	Dusky grouper	[71]	Phenanthrene	0.47–3.76 mg L^−1^	96 h
*Nodipecten nodosus*	Lions-paw scallop	[106]	Phenanthrene	50 and 200 µg L^−1^	96 h
*Sebastiscus marmoratus*	Sea ruffle	[10]	Phenanthrene	0.06–6 μg L^−1^	50 days
*Lates calcarifer*	Barramundi	[73]	Pyrene	1–275 nM	24 h
*Sebastiscus marmoratus*	Sea ruffle	[107]	Pyrene	10.2–102 mg L^−1^	5 days
*Mytilus galloprovincialis*	Mediterranean mussel	[82]	Anthracene	0.05, 0.15, 0.4 µg L^−1^	8 days

* indicates the exposure tests followed by “clearance” of, respectively, 144 h and 14 days.

## Data Availability

Data sharing not applicable.

## References

[1] Bordier M., Uea-Anuwong T., Binot A., Hendrikx P., Goutard F.L. (2020). Characteristics of one health surveillance systems: A systematic literature review. Prev. Vet. Med..

[2] Destoumieux-Garzón D., Mavingui P., Boetsch G., Boissier J., Darriet F., Duboz P., Fritsch C., Giraudoux P., Le Roux F., Morand S. (2018). The one health concept: 10 years old and a long road ahead. Front. Vet. Sci..

[3] Asagbra M.C., Adebayo A.S., Anumudu C.I., Ugwumba O.A., Ugwumba A.A.A. (2015). Polycyclic aromatic hydrocarbons in water, sediment and fish from the Warri River at Ubeji, Niger Delta, Nigeria. Afr. J. Aquat. Sci..

[4] Achten C., Hofmann T. (2009). Native Polycyclic Aromatic Hydrocarbons (PAH) in coals—A hardly recognized source of environmental contamination. Sci. Total Environ..

[5] Naudin G., Bastien P., Mezzache S., Trehu E., Bourokba N., Appenzeller B.M.R., Soeur J., Bornschlögl T. (2019). Human pollution exposure correlates with accelerated ultrastructural degradation of hair fibers. Proc. Natl. Acad. Sci. USA.

[6] Ranjbar Jafarabadi A., Mashjoor S., Riyahi Bakhtiari A., Jadot C. (2020). Dietary intake of polycyclic aromatic hydrocarbons (PAHs) from coral reef fish in the Persian Gulf—Human health risk assessment. Food Chem..

[7] Li R., Hua P., Zhang J., Krebs P. (2020). Effect of anthropogenic activities on the occurrence of polycyclic aromatic hydrocarbons in aquatic suspended particulate matter: Evidence from Rhine and Elbe Rivers. Water Res..

[8] Ma W.L., Zhu F.J., Liu L.Y., Jia H.L., Yang M., Li Y.F. (2019). PAHs in Chinese atmosphere: Gas/particle partitioning. Sci. Total Environ..

[9] Moon H.B., Kim H.S., Choi M., Choi H.G. (2010). Intake and Potential Health Risk of Polycyclic Aromatic Hydrocarbons Associated with Seafood Consumption in Korea from 2005 to 2007. Arch. Environ. Contam. Toxicol..

[10] Sun S.J., Zhao Z.B., Li B., Ma L.X., Fu D.L., Sun X.Z., Thapa S., Shen J.M., Qi H., Wu Y.N. (2019). Occurrence, composition profiles and risk assessment of polycyclic aromatic hydrocarbons in municipal sewage sludge in China. Environ. Pollut..

[11] Sun L., Zuo Z., Luo H., Chen M., Zhong Y., Chen Y., Wang C. (2011). Chronic exposure to phenanthrene influences the Spermatogenesis of Male *Sebastiscus marmoratus*: U-Shaped effects and the reason for them. Environ. Sci. Technol..

[12] Gu Y.G., Ke C.L., Liu Q., Lin Q. (2016). Polycyclic aromatic hydrocarbons (PAHs) in sediments of Zhelin Bay, the largest mariculture base on the eastern Guangdong coast, South China: Characterization and risk implications. Mar. Pollut. Bull..

[13] D’Adamo P., Fassone L., Gedeon A., Janssen E.A.M., Bione S., Bolhuis P.A., Barth P.G., Wilson M., Haan E., Örstavik K.H. (1997). The X-Linked Gene G4.5 Is Responsible for Different Infantile Dilated Cardiomyopathies. Am. J. Hum. Genet..

[14] Wang Q., Yang H., Liu B., Wang X. (2012). Toxic effects of benzo[a]pyrene (Bap) and Aroclor1254 on embryogenesis, larval growth, survival and metamorphosis of the bivalve Meretrix meretrix. Ecotoxicology.

[15] Abdel-Shafy H.I., Mansour M.S.M. (2016). A review on polycyclic aromatic hydrocarbons: Source, environmental impact, effect on human health and remediation. Egypt. J. Pet..

[16] Zhang L., Cao Y., Colella N.S., Liang Y., Brédas J.L., Houk K.N., Briseno A.L. (2015). Unconventional, Chemically Stable, and Soluble Two-Dimensional Angular Polycyclic Aromatic Hydrocarbons: From Molecular Design to Device Applications. Acc. Chem. Res..

[17] Oleagoitia M.B.Z., Manterola A.L., Maurolagoitia J.I., de Dicastillo M.D.M.L., Álvarez J., Barandiaran M.A., Loibide A.I., Santa-Marina L. (2019). Polycyclic aromatic hydrocarbons (PAHs) in air associated with particles PM 2.5 in the Basque Country (Spain). Air Qual. Atmos. Health.

[18] Syed J.H., Iqbal M., Zhong G., Katsoyiannis A., Yadav I.C., Li J., Zhang G. (2017). Polycyclic aromatic hydrocarbons (PAHs) in Chinese forest soils: Profile composition, spatial variations and source apportionment. Sci. Rep..

[19] Bansal V., Kim K.H. (2015). Review of PAH contamination in food products and their health hazards. Environ. Int..

[20] Karlsson K., Viklander M. (2008). Polycyclic Aromatic Hydrocarbons (PAH) in Water and Sediment from Gully Pots. Water Air Soil Pollut..

[21] Lourenço R.A., Taniguchi S., da Silva J., Gallotta F.D.C., Bícego M.C. (2021). Polycyclic aromatic hydrocarbons in marine mammals: A review and synthesis. Mar. Pollut. Bull..

[22] Pérez-Cadahía B., Laffon B., Pásaro E., Méndez J. (2004). Evaluation of PAH Bioaccumulation and DNA Damage in Mussels (*Mytilus galloprovincialis*) Exposed to Spilled Prestige Crude Oil. Comp. Biochem. Physiol.-C Toxicol. Pharmacol..

[23] Bandowe B.A.M., Bigalke M., Boamah L., Nyarko E., Saalia F.K., Wilcke W. (2014). Polycyclic Aromatic Compounds (PAHs and Oxygenated PAHs) and Trace Metals in Fish Species from Ghana (West Africa): Bioaccumulation and Health Risk Assessment. Environ. Int..

[24] Quiroz R., Grimalt J.O., Fernández P. (2010). Toxicity Assessment of Polycyclic Aromatic Hydrocarbons in Sediments from European High Mountain Lakes. Ecotoxicol. Environ. Saf..

[25] Dural M., Göksu M.Z.L., Özak A.A. (2007). Investigation of Heavy Metal Levels in Economically Important Fish Species Captured from the Tuzla Lagoon. Food Chem..

[26] Dickey R.W. (2012). FDA Risk Assessment of Seafood Contamination after the BP Oil Spill. Environ. Health Perspect..

[27] Magalhães D.D.P., Ferrão Filho A.D.S. (2008). A ecotoxicologia como ferramenta no biomonitoramento de ecossistemas aquáticos. Oecol. Bras..

[28] Castaño A., Sanchez P., Llorente M.T., Carballo M., De La Torre A., Muñoz M.J. (2000). The use of alternative systems for the ecotoxicological screening of complex mixtures on fish populations. Sci. Total Environ..

[29] Barone G., Storelli A., Garofalo R., Busco V.P., Quaglia N.C., Centrone G., Storelli M.M. (2015). Assessment of mercury and cadmium via seafood consumption in Italy: Estimated dietary intake (EWI) and target hazard quotient (THQ). Food Addit. Contam. Part A.

[30] R: The R Project for Statistical Computing. https://www.r-project.org/.

[31] Luís de Sá Salomão A., Hauser-Davis R.A., Marques M. (2020). Critical Knowledge Gaps and Relevant Variables Requiring Consideration When Performing Aquatic Ecotoxicity Assays. Ecotoxicol. Environ. Saf..

[32] Hodson P.V. (2017). The Toxicity to Fish Embryos of PAH in Crude and Refined Oils. Arch. Environ. Contam. Toxicol..

[33] Govers H., Ruepert C., Aiking H. (1984). Quantitative Structure-Activity Relationships for Polycyclic Aromatic Hydrocarbons: Correlation between Molecular Connectivity, Physico-Chemical Properties, Bioconcentration and Toxicity in Daphnia Pulex. Chemosphere.

[34] Carls M.G., Meador J.P. (2009). A Perspective on the Toxicity of Petrogenic PAHs to Developing Fish Embryos Related to Environmental Chemistry. Hum. Ecol. Risk Assess..

[35] Harvey R.G. (1998). Environmental Chemistry of PAHs. PAHs and Related Compounds.

[36] Logan D.T. (2007). Perspective on Ecotoxicology of PAHs to Fish. Hum. Ecol. Risk Assess..

[37] Wilke B.M., Riepert F., Koch C., Kühne T. (2008). Ecotoxicological Characterization of Hazardous Wastes. Ecotoxicol. Environ. Saf..

[38] Díaz-Cruz M.S., Barceló D. (2009). Chemical Analysis and Ecotoxicological Effects of Organic UV-Absorbing Compounds in Aquatic Ecosystems. TrAC Trends Anal. Chem..

[39] Escher B.I., Hermens J.L.M. (2002). Modes of Action in Ecotoxicology: Their Role in Body Burdens, Species Sensitivity, QSARs, and Mixture Effects. Environ. Sci. Technol..

[40] PubChem. https://pubchem.ncbi.nlm.nih.gov/.

[41] Nałȩcz-Jawecki G., Sawicki J. (1999). Spirotox—A New Tool for Testing the Toxicity of Volatile Compounds. Chemosphere.

[42] Ololade I.A., Arogunrerin I.A., Oladoja N.A., Ololade O.O., Alabi A.B. (2021). Concentrations and Toxic Equivalency of Polycyclic Aromatic Hydrocarbons (PAHs) and Polychlorinated Biphenyl (PCB) Congeners in Groundwater around Waste Dumpsites in South-West Nigeria. Arch. Environ. Contam. Toxicol..

[43] Zacchi F.L., de Lima D., Flores-Nunes F., Mattos J.J., Lüchmann K.H., de Miranda Gomes C.H.A., Bícego M.C., Taniguchi S., Sasaki S.T., Dias Bainy A.C. (2017). Transcriptional Changes in Oysters Crassostrea Brasiliana Exposed to Phenanthrene at Different Salinities. Aquat. Toxicol..

[44] Le Dû-Lacoste M., Akcha F., Dévier M.H., Morin B., Burgeot T., Budzinski H. (2013). Comparative Study of Different Exposure Routes on the Biotransformation and Genotoxicity of PAHs in the Flatfish Species, Scophthalmus Maximus. Environ. Sci. Pollut. Res..

[45] Grintzalis K., Georgiou C.D., Dailianis S. (2012). Total Thiol Redox Status as a Potent Biomarker of PAH-Mediated Effects on Mussels. Mar. Environ. Res..

[46] Magara G., Elia A.C., Syberg K., Khan F.R. (2018). Single Contaminant and Combined Exposures of Polyethylene Microplastics and Fluoranthene: Accumulation and Oxidative Stress Response in the Blue Mussel, Mytilus Edulis. J. Toxicol. Environ. Health-Part A Curr. Issues.

[47] Speciale A., Zena R., Calabrò C., Bertuccio C., Aragona M., Saija A., Trombetta D., Cimino F., lo Cascio P. (2018). Experimental Exposure of Blue Mussels (*Mytilus galloprovincialis*) to High Levels of Benzo[a]Pyrene and Possible Implications for Human Health. Ecotoxicol. Environ. Saf..

[48] Barranger A., Langan L.M., Sharma V., Rance G.A., Aminot Y., Weston N.J., Akcha F., Moore M.N., Arlt V.M., Khlobystov A.N. (2019). Antagonistic Interactions between Benzo[a]Pyrene and Fullerene (C60) in Toxicological Response of Marine Mussels. Nanomaterials.

[49] Moore F., Akhbarizadeh R., Keshavarzi B., Khabazi S., Lahijanzadeh A., Kermani M. (2015). Ecotoxicological Risk of Polycyclic Aromatic Hydrocarbons (PAHs) in Urban Soil of Isfahan Metropolis, Iran. Environ. Monit. Assess..

[50] Witter A.E., Nguyen M.H., Baidar S., Sak P.B. (2014). Coal-Tar-Based Sealcoated Pavement: A Major PAH Source to Urban Stream Sediments. Environ. Pollut..

[51] Fisher T.T., Law R.J., Rumney H.S., Kirby M.F., Kelly C. (2011). Towards a Scheme of Toxic Equivalency Factors (TEFs) for the Acute Toxicity of PAHs in Sediment. Ecotoxicol. Environ. Saf..

[52] Baird W.M., Hooven L.A., Mahadevan B. (2005). Carcinogenic Polycyclic Aromatic Hydrocarbon-DNA Adducts and Mechanism of Action. Environ. Mol. Mutagen..

[53] Song Y., Nahrgang J., Tollefsen K.E. (2019). Transcriptomic Analysis Reveals Dose-Dependent Modes of Action of Benzo(a)Pyrene in Polar Cod (*Boreogadus saida*). Sci. Total Environ..

[54] Hylland K. (2007). Polycyclic Aromatic Hydrocarbon (PAH) Ecotoxicology in Marine Ecosystems. J. Toxicol. Environ. Health.

[55] Hasue F.M., Passos M.J.D.A.C.R., dos Santos T.D.C.A., Rocha A.J.D.S., Vignardi C.P., Sartorio P.V., Gomes V., van Ngan P. (2013). Assessment of Genotoxicity and Depuration of Anthracene in the Juvenile Coastal Fish Trachinotus Carolinus Using the Comet Assay. Braz. J. Oceanogr..

[56] Palanikumar L., Kumaraguru A.K., Ramakritinan C.M., Anand M. (2012). Biochemical Response of Anthracene and Benzo [a] Pyrene in Milkfish Chanos Chanos. Ecotoxicol. Environ. Saf..

[57] Abramochkin D.V., Kompella S.N., Shiels H.A. (2021). Phenanthrene Alters the Electrical Activity of Atrial and Ventricular Myocytes of a Polar Fish, the Navaga Cod. Aquat. Toxicol..

[58] Li R., Zuo Z., Chen D., He C., Chen R., Chen Y., Wang C. (2011). Inhibition by Polycyclic Aromatic Hydrocarbons of ATPase Activities in *Sebastiscus marmoratus* Larvae in Relationship with the Development of Early Life Stages. Mar. Environ. Res..

[59] Zena R., Speciale A., Calabrò C., Calò M., Palombieri D., Saija A., Cimino F., Trombetta D., lo Cascio P. (2015). Exposure of Sea Bream (*Sparus aurata*) to Toxic Concentrations of Benzo[a]Pyrene: Possible Human Health Effect. Ecotoxicol. Environ. Saf..

[60] Honda M., Suzuki N. (2020). Toxicities of Polycyclic Aromatic Hydrocarbons for Aquatic Animals. Int. J. Environ. Res. Public Health.

[61] Wilhelm Filho D., Tribess T., Gáspari C., Claudio F.D., Torres M.A., Magalhães A.R.M. (2001). Seasonal Changes in Antioxidant Defenses of the Digestive Gland of the Brown Mussel (*Perna perna*). Aquaculture.

[62] Maria V.L., Gomes T., Barreira L., Bebianno M.J. (2013). Impact of Benzo(a)Pyrene, Cu and Their Mixture on the Proteomic Response of *Mytilus galloprovincialis*. Aquat. Toxicol..

[63] Saeed T., Ali L.N., Al-Bloushi A., Al-Hashash H., Al-Bahloul M., Al-Khabbaz A., Al-Khayat A. (2011). Effect of Environmental Factors on Photodegradation of Polycyclic Aromatic Hydrocarbons (PAHs) in the Water-Soluble Fraction of Kuwait Crude Oil in Seawater. Mar. Environ. Res..

[64] Vieira L.R., Guilhermino L. (2012). Multiple Stress Effects on Marine Planktonic Organisms: Influence of Temperature on the Toxicity of Polycyclic Aromatic Hydrocarbons to Tetraselmis Chuii. J. Sea Res..

[65] McCloskey J.T., Oris J.T. (1993). Effect of Anthracene and Solar Ultraviolet Radiation Exposure on Gill ATPase and Selected Hematologic Measurements m the Bluegill Sunfish (*Lepomis macrochirus*). Aquat. Toxicol..

[66] Serafin J., Guffey S.C., Bosker T., Griffitt R.J., de Guise S., Perkins C., Szuter M., Sepúlveda M.S. (2019). Combined Effects of Salinity, Temperature, Hypoxia, and Deepwater Horizon Oil on Fundulus Grandis Larvae. Ecotoxicol. Environ. Saf..

[67] Brinkmann M., Blenkle H., Salowsky H., Bluhm K., Schiwy S., Tiehm A., Hollert H. (2014). Genotoxicity of Heterocyclic PAHs in the Micronucleus Assay with the Fish Liver Cell Line RTL-W1. PLoS ONE.

[68] Olson G.M., Meyer B.M., Portier R.J. (2016). Assessment of the Toxic Potential of Polycyclic Aromatic Hydrocarbons (PAHs) Affecting Gulf Menhaden (*Brevoortia patronus*) Harvested from Waters Impacted by the BP Deepwater Horizon Spill. Chemosphere.

[69] Mansour C., Guardiola F.A., Esteban M.Á., Mosbahi D.S. (2017). Combination of Polycyclic Aromatic Hydrocarbons and Temperature Exposure: In Vitro Effects on Immune Response of European Clam (*Ruditapes decussatus*). Fish Shellfish Immunol..

[70] Ramachandran S.D., Sweezey M.J., Hodson P.V., Boudreau M., Courtenay S.C., Lee K., King T., Dixon J.A. (2006). Influence of Salinity and Fish Species on PAH Uptake from Dispersed Crude Oil. Mar. Pollut. Bull..

[71] De Campos M.F., lo Nostro F.L., da Cuña R.H., Moreira R.G. (2018). Endocrine Disruption of Phenanthrene in the Protogynous Dusky Grouper Epinephelus Marginatus (*Serranidae: Perciformes*). Gen. Comp. Endocrinol..

[72] Almeida J.R., Gravato C., Guilhermino L. (2012). Biological Parameters towards Polycyclic Aromatic Hydrocarbons Pollution: A Study with Dicentrarchus Labrax L. Exposed to the Model Compound Benzo(a)Pyrene. Water Air Soil Pollut..

[73] Guven O., Bach L., Munk P., Dinh K.V., Mariani P., Nielsen T.G. (2018). Microplastic Does Not Magnify the Acute Effect of PAH Pyrene on Predatory Performance of a Tropical Fish (*Lates calcarifer*). Aquat. Toxicol..

[74] Dailianis S., Tsarpali V., Melas K., Karapanagioti H.K., Manariotis I.D. (2014). Aqueous Phenanthrene Toxicity after High-Frequency Ultrasound Degradation. Aquat. Toxicol..

[75] Page D.S., Chapman P.M., Landrum P.F., Neff J., Elston R. (2012). A Perspective on the Toxicity of Low Concentrations of Petroleum-Derived Polycyclic Aromatic Hydrocarbons to Early Life Stages of Herring and Salmon. Hum. Ecol. Risk Assess. Int. J..

[76] Thorpe K.L., Gross-Sorokin M., Johnson I., Brighty G., Tyler C.R. (2006). An Assessment of the Model of Concentration Addition for Predicting the Estrogenic Activity of Chemical Mixtures in Wastewater Treatment Works Effluents. Environ. Health Perspect..

[77] Glomstad B., Altin D., Sørensen L., Liu J., Jenssen B.M., Booth A.M. (2016). Carbon Nanotube Properties Influence Adsorption of Phenanthrene and Subsequent Bioavailability and Toxicity to Pseudokirchneriella Subcapitata. Environ. Sci. Technol..

[78] Dale K., Yadetie F., Müller M.B., Pampanin D.M., Gilabert A., Zhang X., Tairova Z., Haarr A., Lille-Langøy R., Lyche J.L. (2020). Proteomics and Lipidomics Analyses Reveal Modulation of Lipid Metabolism by Perfluoroalkyl Substances in Liver of Atlantic Cod (*Gadus morhua*). Aquat. Toxicol..

[79] Farkas J., Bergum S., Nilsen E.W., Olsen A.J., Salaberria I., Ciesielski T.M., Baczek T., Konieczna L., Salvenmoser W., Jenssen B.M. (2015). The Impact of TiO2 Nanoparticles on Uptake and Toxicity of Benzo(a)Pyrene in the Blue Mussel (*Mytilus edulis*). Sci. Total Environ..

[80] Song Q., Chen H., Li Y., Zhou H., Han Q., Diao X. (2016). Toxicological Effects of Benzo(a)Pyrene, DDT and Their Mixture on the Green Mussel Perna Viridis Revealed by Proteomic and Metabolomic Approaches. Chemosphere.

[81] Wang H., Cui L., Cheng H., Zhang Y., Diao X., Wang J. (2017). Comparative Studies on the Toxicokinetics of Benzo[a]Pyrene in Pinctada Martensii and Perna Viridis. Bull. Environ. Contam. Toxicol..

[82] Badreddine S., Abdelhafidh K., Dellali M., Mahmoudi E., Sheehan D., Hamouda B. (2017). The Effects of Anthracene on Biochemical Responses of Mediterranean Mussels *Mytilus galloprovincialis*. Chem. Ecol..

[83] FAO (1998). Fishery status for capture production. FAO Yearbook for 1996.

[84] Zanaty M.I., Sawada N., Kitani Y., Nassar H.F., Mahmoud H.M., Hayakawa K., Sekiguchi T., Ogiso S., Tabuchi Y., Urata M. (2020). Influence of Benz[a]Anthracene on Bone Metabolism and on Liver Metabolism in Nibbler Fish, Girella Punctata. Int. J. Environ. Res. Public Health.

[85] Tian S., Pan L., Zhang H. (2014). Identification of a CYP3A-like Gene and CYPs MRNA Expression Modulation Following Exposure to Benzo[a]Pyrene in the Bivalve Mollusk *Chlamys farreri*. Mar. Environ. Res..

[86] Tian S., Pan L., Sun X. (2013). An Investigation of Endocrine Disrupting Effects and Toxic Mechanisms Modulated by Benzo[a]Pyrene in Female Scallop *Chlamys farreri*. Aquat. Toxicol..

[87] Tian S., Pan L., Tao Y., Sun X. (2015). Environmentally Relevant Concentrations of Benzo[a]Pyrene Affect Steroid Levels and Affect Gonad of Male Scallop *Chlamys farreri*. Ecotoxicol. Environ. Saf..

[88] Li L., Ping X., Li Z., Xv G., Wang C., Jiang M. (2021). Effects of Oxidation Defense System Exposure to Benzo(a)Pyrene on CYP450 Gene Expression and EROD Activity in Crassostrea Gigas and Mytilus Coruscus. Environ. Pollut. Bioavailab..

[89] Yadetie F., Brun N.R., Vieweg I., Nahrgang J., Karlsen O.A., Goksøyr A. (2021). Transcriptome Responses in Polar Cod (*Boreogadus saida*) Liver Slice Culture Exposed to Benzo[a]Pyrene and Ethynylestradiol: Insights into Anti-Estrogenic Effects. Toxicol. Vitr..

[90] Ren X., Pan L., Wang L. (2014). Metabolic Enzyme Activities, Metabolism-Related Genes Expression and Bioaccumulation in Juvenile White Shrimp Litopenaeus Vannamei Exposed to Benzo[a]Pyrene. Ecotoxicol. Environ. Saf..

[91] García-Tavera J.L., Valdés-Lozano D., Poblete-Naredo I., Albores-Medina A., Zapata-Pérez O. (2013). Bile Benzo[a]Pyrene Concentration and Hepatic CYP1A Induction in Hypoxic Adult Tilapia (*Oreochromis niloticus*). Chemosphere.

[92] Strobel A., Mark F.C., Segner H., Burkhardt-Holm P. (2018). Expression of Aryl Hydrocarbon Receptor–Regulated Genes and Superoxide Dismutase in the Antarctic Eelpout Pachycara Brachycephalum Exposed to Benzo[a]Pyrene. Environ. Toxicol. Chem..

[93] Soltani T., Safahieh A., Zolgharnain H., Matroodi S. (2019). Interactions of Oxidative DNA Damage and CYP1A Gene Expression with the Liver Enzymes in Klunzinger’s Mullet Exposed to Benzo[a]Pyrene. Toxicol. Rep..

[94] Wen J., Pan L. (2016). Short-Term Exposure to Benzo[a]Pyrene Causes Oxidative Damage and Affects Haemolymph Steroid Levels in Female Crab Portunus Trituberculatus. Environ. Pollut..

[95] Wen J., Pan L. (2015). Short-Term Exposure to Benzo[a]Pyrene Disrupts Reproductive Endocrine Status in the Swimming Crab Portunus Trituberculatus. Comp. Biochem. Physiol. Part C Toxicol. Pharmacol..

[96] Liu D., Pan L., Li Z., Cai Y., Miao J. (2014). Metabolites Analysis, Metabolic Enzyme Activities and Bioaccumulation in the Clam Ruditapes Philippinarum Exposed to Benzo[a]Pyrene. Ecotoxicol. Environ. Saf..

[97] Wang H., Pan L., Si L., Ji R., Cao Y. (2021). Effects of Nrf2-Keap1 Signaling Pathway on Antioxidant Defense System and Oxidative Damage in the Clams Ruditapes Philippinarum Exposure to PAHs. Environ. Sci. Pollut. Res..

[98] Wang H., Pan L., Xu R., Miao J., Si L., Pan L. (2018). Comparative Transcriptome Analysis between the Short-Term Stress and Long-Term Adaptation of the Ruditapes Philippinarum in Response to Benzo[a]Pyrene. Aquat. Toxicol..

[99] Zhang H., Zhao L. (2017). In Fl Uence of Sublethal Doses of Acetamiprid and Halosulfuron-Methyl on Metabolites of Zebra Fi Sh (*Brachydanio rerio*). Aquat. Toxicol..

[100] Woo S.J., Chung J.K. (2020). Cytochrome P450 1 Enzymes in Black Rockfish, Sebastes Schlegelii: Molecular Characterization and Expression Patterns after Exposure to Benzo[a]Pyrene. Aquat. Toxicol..

[101] Zacchino V., Centoducati G., Narracci M., Selvaggi M., Santacroce M.P. (2013). Effects of Benzo[a]Pyrene on Gilthead Sea Breams (Sparusaurata L.) Hepatocytes Exposeidn Vitroto Short and Long Term Trials. Ital. J. Anim. Sci..

[102] Da Silva Rocha A.J., Santos T.C.A., Gomes V., Bícego M.C., Barbosa A.C.R.d.A., Passos M.J.d.A.C.R., Hasue F.M., van Ngan P. (2012). Assessment of Trophic Transfer of Benzo(a)Pyrene Genotoxicity from the Post-Larval Pink Shrimp F. Brasiliensis to the Juvenile Florida Pompano T. Carolinus. Environ. Toxicol. Pharmacol..

[103] Deng X., Pan L., Cai Y., Jin Q. (2016). Transcriptomic Changes in the Ovaries of Scallop *Chlamys farreri* Exposed to Benzo[a]Pyrene. Genes Genom..

[104] Xu R., Pan L., Yang Y., Zhou Y., Li D. (2020). Temporal Transcriptome Analysis in Female Scallop *Chlamys farreri*: First Molecular Insights into the Disturbing Mechanism on Lipid Metabolism of Reproductive-Stage Dependence under Benzo[a]Pyrene Exposure. Sci. Total Environ..

[105] Lüchmann K.H., Dafre A.L., Trevisan R., Craft J.A., Meng X., Mattos J.J., Zacchi F.L., Dorrington T.S., Schroeder D.C., Bainy A.C.D. (2014). A Light in the Darkness: New Biotransformation Genes, Antioxidant Parameters and Tissue-Specific Responses in Oysters Exposed to Phenanthrene. Aquat. Toxicol..

[106] Piazza R.S., Trevisan R., Flores-Nunes F., Toledo-Silva G., Wendt N., Mattos J.J., Lima D., Taniguchi S., Sasaki S.T., Mello Á.C.P. (2016). Exposure to Phenanthrene and Depuration: Changes on Gene Transcription, Enzymatic Activity and Lipid Peroxidation in Gill of Scallops Nodipecten Nodosus. Aquat. Toxicol..

[107] He C., Zuo Z., Shi X., Sun L., Wang C. (2012). Pyrene Exposure Influences the Thyroid Development of *Sebastiscus marmoratus* Embryos. Aquat. Toxicol..

[108] Ali N. (2019). Polycyclic Aromatic Hydrocarbons (PAHs) in Indoor Air and Dust Samples of Different Saudi Microenvironments; Health and Carcinogenic Risk Assessment for the General Population. Sci. Total Environ..

[109] Gravato C., Almeida J.R., Silva C., Oliveira C., Soares A.M.V.M. (2014). Using a Multibiomarker Approach and Behavioural Responses to Assess the Effects of Anthracene in Palaemon Serratus. Aquat. Toxicol..

[110] Olatunji O.S., Fatoki O.S., Opeolu B.O., Ximba B.J. (2015). Benzo[a]Pyrene and Benzo[k]Fluoranthene in Some Processed Fish and Fish Products. Int. J. Environ. Res. Public Health.

[111] U.S. Environmental Protection Agency (1992). Report on the Ecological Risk Assessment Guidelines Strategic Planning Workshop.

[112] Suter G.W. (1993). A Critique of Ecosystem Health Concepts and Indexes. Environ. Toxicol. Chem..

[113] Van Leeuwen C.J., Verhaar H.J.M., Hermens J.L.M. (2008). Quality Criteria and Risk Assessment for Mixtures of Chemicals in the Aquatic Environment. Hum. Ecol. Risk Assess. Int. J..

[114] Cao Z., Liu J., Luan Y., Li Y., Ma M., Xu J., Han S. (2010). Distribution and Ecosystem Risk Assessment of Polycyclic Aromatic Hydrocarbons in the Luan River, China. Ecotoxicology.

